# Microbial synthesis of Pd/Fe_3_O_4_, Au/Fe_3_O_4_ and PdAu/Fe_3_O_4_ nanocomposites for catalytic reduction of nitroaromatic compounds

**DOI:** 10.1038/srep13515

**Published:** 2015-08-27

**Authors:** Ya Tuo, Guangfei Liu, Bin Dong, Jiti Zhou, Aijie Wang, Jing Wang, Ruofei Jin, Hong Lv, Zeou Dou, Wenyu Huang

**Affiliations:** 1 Key Laboratory of Industrial Ecology and Environmental Engineering, Ministry of Education, School of Environmental Science and Technology, Dalian University of Technology, Dalian, 116024, China; 2 State Key Laboratory of Urban Water Resource and Environment, Harbin Institute of Technology, Harbin, 150090, China

## Abstract

Magnetically recoverable noble metal nanoparticles are promising catalysts for chemical reactions. However, the chemical synthesis of these nanocatalysts generally causes environmental concern due to usage of toxic chemicals under extreme conditions. Here, Pd/Fe_3_O_4_, Au/Fe_3_O_4_ and PdAu/Fe_3_O_4_ nanocomposites are biosynthesized under ambient and physiological conditions by *Shewanella oneidensis* MR-1. Microbial cells firstly transform akaganeite into magnetite, which then serves as support for the further synthesis of Pd, Au and PdAu nanoparticles from respective precursor salts. Surface-bound cellular components and exopolysaccharides not only function as shape-directing agent to convert some Fe_3_O_4_ nanoparticles to nanorods, but also participate in the formation of PdAu alloy nanoparticles on magnetite. All these three kinds of magnetic nanocomposites can catalyze the reduction of 4-nitrophenol and some other nitroaromatic compounds by NaBH_4_. PdAu/Fe_3_O_4_ demonstrates higher catalytic activity than Pd/Fe_3_O_4_ and Au/Fe_3_O_4_. Moreover, the magnetic nanocomposites can be easily recovered through magnetic decantation after catalysis reaction. PdAu/Fe_3_O_4_ can be reused in at least eight successive cycles of 4-nitrophenol reduction. The biosynthesis approach presented here does not require harmful agents or rigorous conditions and thus provides facile and environmentally benign choice for the preparation of magnetic noble metal nanocatalysts.

Noble metal catalysts have drawn considerable attention due to their unique physicochemical properties, which lead to versatile applications ranging from catalyzing organic transformation reactions to chemical/biological sensing, surface-enhanced Raman scattering, fuel cells, and hydrogen storage etc^1^,^2^,^3^,^4^. However, conventional approaches to synthesize these nanoparticles are generally accompanied by the use of toxic or dangerous chemicals and high consumption of energy under extreme conditions, which cause great environmental concern. Interestingly, different microorganisms have been found capable of synthesizing inorganic nanoparticles under mild conditions, which provides potential “green” alternatives to traditional chemical and physical methods^5^,^6^.

The typical metal-reducing bacterium *Shewanella oneidensis* has attracted a lot of attention in recent years due to its considerable capacity for electricity generation and pollutants removal. Moreover, it was also found capable of synthesizing and interacting with various nanomaterials, which broadens the knowledge of bacteria-nanomaterial interface under natural or laboratory conditions^7^,^8^. The respiration diversity of *S. oneidensis* has been applied for bioreduction of various metals and metalloids, such as uranium, chromium, technetium, plutonium, neptunium, gold, silver, palladium, vanadate, iodate, selenite and tellurite etc., some of which can be reduced to their elementary states^9^,^10^. Suresh *et al*. successfully fabricated discrete spherical Au nanoparticles having low biotoxicity with *S. oneidensis*^10^. By using *S. oneidensis* cells as reducing agents and supports, bio-Pd and bio-PdAu nanoparticles (i.e. monometallic Pd and alloyed PdAu nanoparticles located on cells, respectively) were synthesized and applied for reductive dechlorination of pollutants through collaboration between microbial cells and noble metal catalysts^11^,^12^. However, the negative and toxic effects of the “metal armor” on normal metabolism and proliferation activities of microbial cells may hinder the long-term or repeated using of these palladized cells. On the other hand, some researchers removed *Shewanella* and other bacterial cells after biosynthesis processes through calcination or pyrolysis and made use of the biotemplated nanoparticles in a purely chemical or electrochemical way^13^,^14^,^15^.

Due to their large surface areas, metal nanocatalysts without a suitable support could easily aggregate in solution, which always results in remarkable reduction of their catalytic activities. In addition, it is difficult to recycle these nanocatalysts from reaction solution because of their small sizes. Magnetite (Fe_3_O_4_) is an ideal support, which is easy to prepare and has a very active surface for the adsorption/immobilization of metals and ligands. It can not only prevent the aggregation of metal nanoparticles, but also facilitate the recycle of nanocatalysts through magnetic separation^16^,^17^. *Shewanella* strains are well-known to play important roles in biogeochemical cycles of iron and can biologically transform iron oxyhydroxides like ferrihydrite and akaganeite into nanoscaled magnetite under normal biomineralization conditions^7^,^18^,^19^. However, it is surprising to find that no study has been carried out for the biological preparation of magnetically recoverable noble metal nanocatalysts using *S. oneidensis*.

In the present work, we demonstrate that monometallic Pd, Au and bimetallic PdAu alloy nanoparticles can be produced on biofabricated magnetite under ambient conditions with *S. oneidensis* MR-1. Organic substances like cellular components and exopolysaccharides, which are generated by MR-1 cells and remain on biogenic magnetite, help the generation and growth of rod-like magnetite and formation of PdAu alloy nanoparticles on magnetite support. The resultant nanocomposites have high catalytic activities towards the reduction of different nitroarenes. The synergistic effect between Pd and Au endows PdAu/Fe_3_O_4_ with superior activity compared with Pd/Fe_3_O_4_ and Au/Fe_3_O_4_.

## Results

### Synthesis and characterization of Fe_3_O_4_-supported noble metal nanocomposites

Using lactate as electron donor, *S. oneidensis* MR-1 can transform non-magnetic akaganeite to magnetic precipitate in 48 h under anaerobic conditions. Transmission electron microscopy (TEM) analysis demonstrates that the size of the formed nanoparticles ranges between 3 and 15 nm (see Supplementary Fig. S1). Data of X-ray diffraction (XRD) analysis of the magnetic nanoparticles match well with the diffractions from metallic face-centered cubic (fcc) Fe_3_O_4_ at 2*θ* = 18.04°, 29.96°, 35.20°, 42.82°, 53.32°, 57.04°, 62.60° and 74.06°, which arise from the (111), (220), (311), (400), (422), (511), (440) and (533) planes (JCPDS 19-0629), respectively (see Supplementary Fig. S2).

Fe_3_O_4_ loaded with monometallic or bimetallic noble metals (Pd or/and Au) were produced through adding Pd(II) or/and Au(III) precursor salt solutions to the water-washed biogenic Fe_3_O_4_ suspension and incubating for another 48 h under anoxic conditions. The final molar ratios of Pd:Fe_3_O_4_ and Au:Fe_3_O_4_ were calculated to be 1:1.6 and 1:1.7 in Pd/Fe_3_O_4_ and Au/Fe_3_O_4_, respectively. For PdAu/Fe_3_O_4_, the final molar ratio of Pd:Au:Fe_3_O_4_ is 1:1.7:1.7. The mole fraction (mol %) of Pd decreases significantly from 38.5 ± 1.8% in Pd/Fe_3_O_4_ to 22.7 ± 1.4% in PdAu/Fe_3_O_4_, whereas no obvious difference in mole fraction of Au was observed between Au/Fe_3_O_4_ (37.0 ± 1.3%) and PdAu/Fe_3_O_4_ (38.6 ± 1.7%).

**Figure 1:**
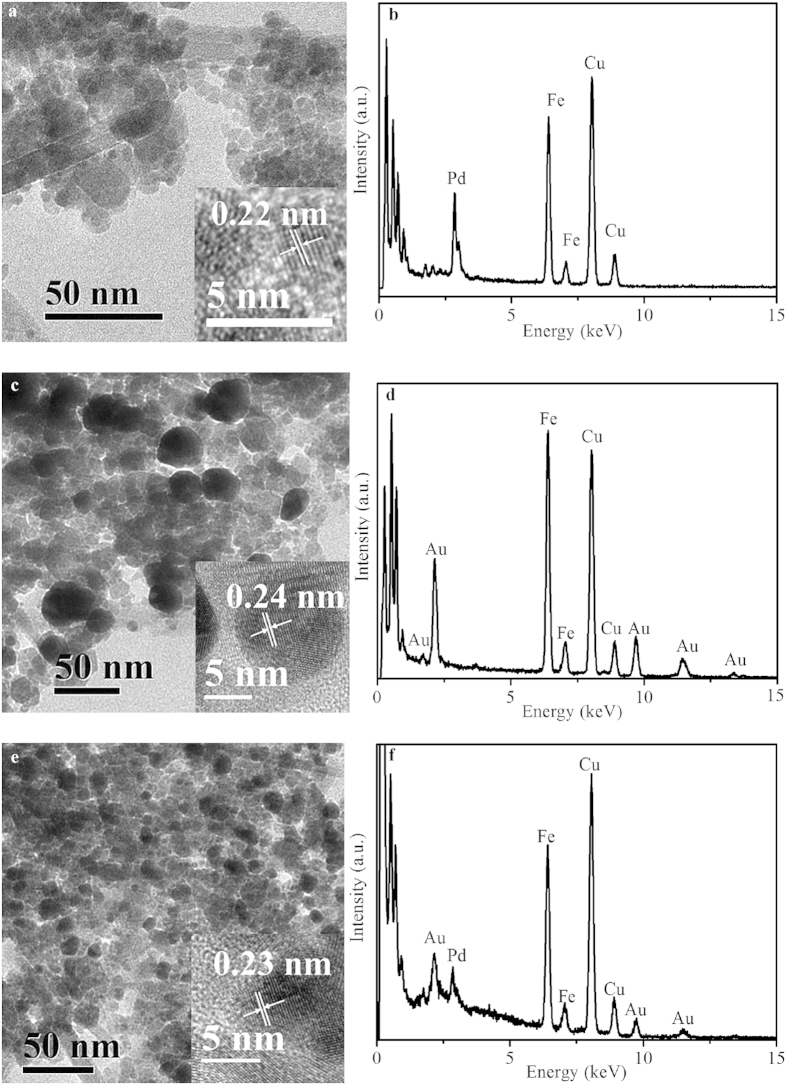
Morphology and element analyses. TEM and HRTEM images (insert) of (**a**) Pd/Fe_3_O_4_, (**c**) Au/Fe_3_O_4_ and (**e**) PdAu/Fe_3_O_4_ obtained through the addition of Pd(II) or/and Au(III) precursor salt solutions to the biogenic Fe_3_O_4_ suspension. The EDX spectra in (**b**,**d**,**f**) correspond to samples of (**a**,**c**,**e**), respectively.

Both nanoparticles and nanorods were observed in the TEM images of the Pd/Fe_3_O_4_, Au/Fe_3_O_4_ and PdAu/Fe_3_O_4_ nanocomposites. For Pd/Fe_3_O_4_, the average diameter of nanoparticle is 5.5 ± 2.2 nm and the nanorods are 100–200 nm in length and 7–17 nm in width (Fig. 1a and also see Supplementary Fig. S3). The high resolution TEM (HRTEM) image reveals that the measured adjacent lattice fringe distance (0.22 nm) corresponds well to the (111) lattice spacing of the fcc Pd. Energy dispersive X-ray (EDX) analysis also confirms the presence of Pd (Fig. 1b). The average diameter of the magnetic nanoparticles increases to 15.4 ± 6.8 nm after the introduction of Au (Fig. 1c and Supplementary Fig. S3), the presence of which was confirmed by the EDX data (Fig. 1d). The Au/Fe_3_O_4_ nanorods are 140–190 nm in length and 10–18 nm in width. The measured *d*-spacing for adjacent lattice planes (0.24 nm) agrees well with the (111) lattice spacing of fcc Au. For PdAu/Fe_3_O_4_, the average diameter of the nanoparticles is around 8.3 ± 3.2 nm, and the nanorods are 200–300 nm in length and 8–18 nm in width. The measured adjacent lattice fringe distance of PdAu nanoparticles is 0.23 nm, which locates between the (111) lattice spacing of fcc Au and that of fcc Pd (Fig. 1e and Supplementary Fig. S3) and suggests the formation of PdAu alloy. The EDX analysis (Fig. 1f) also confirms the presence of both Pd and Au in the nanocomposite. Elemental mapping was conducted to characterize the PdAu/Fe_3_O_4_ nanocomposite (see Supplementary Fig. S4). The uniform color distribution confirms the formation of PdAu alloy structure on magnetite.

HRTEM was utilized to further characterize the rod-like structure appeared after the synthesis of PdAu/Fe_3_O_4_ nanocomposite (see Supplementary Fig. S5). The measured *d*-spacing for the nanorod is 0.16 nm, which corresponds well to the (511) lattice spacing of the fcc Fe_3_O_4_. Notably, the nanorod also acts as support of PdAu alloy nanoparticles, the (200) and (111) lattice spacing of which were found with measured adjacent lattice fringe distance of 0.20 nm and 0.23 nm, respectively.

**Figure 2:**
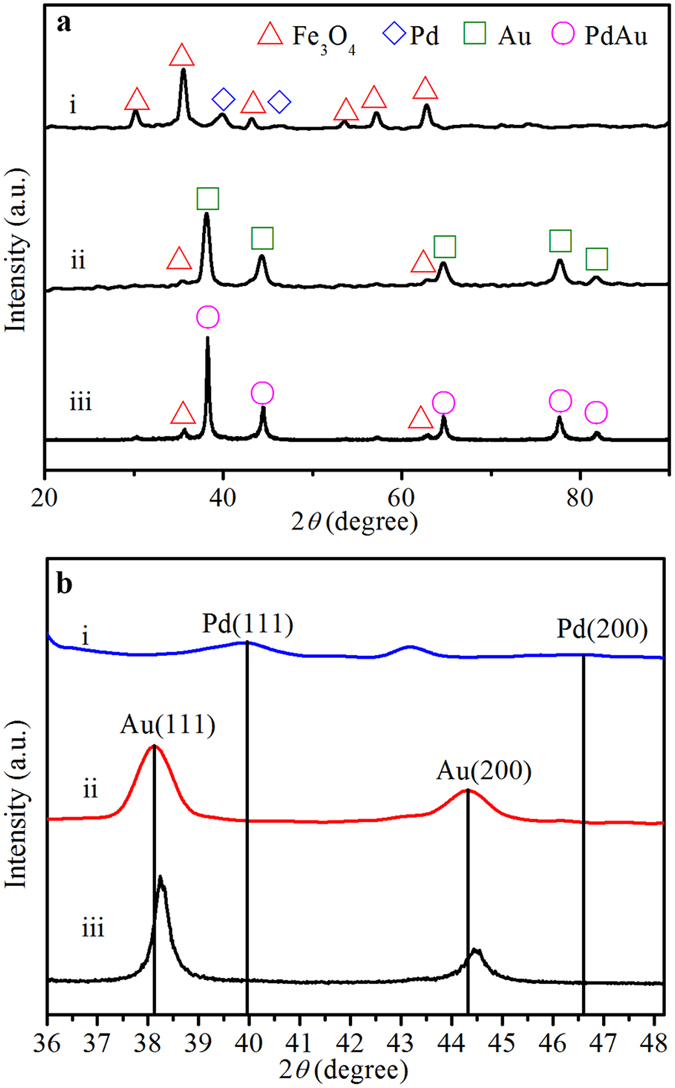
Crystalline structure. (**a**) XRD patterns of (i) Pd/Fe_3_O_4_, (ii) Au/Fe_3_O_4_ and (iii) PdAu/Fe_3_O_4_. (**b**) Magnification of the peaks (111) and (200) in the 2*θ* range of 36–48°.

As shown in Fig. 2a, for Pd/Fe_3_O_4_ nanocomposite, the observed XRD peaks at 2*θ* = 30.18°, 35.54°, 43.18°, 53.58°, 57.16° and 62.78° can be indexed to (220), (311), (400), (422), (511) and (440) planes of fcc Fe_3_O_4_, respectively (JCPDS 19-0629). In addition, the diffraction peaks ascribed to (111) and (200) planes of metallic fcc Pd (JCPDS 46-1043) were clearly observed at 2*θ* = 39.90° and 46.54°, respectively. For Au/Fe_3_O_4_ nanocomposite, the diffraction peaks at 2*θ* = 38.12°, 44.32°, 64.64°, 77.72° and 81.80° can be indexed to the (111), (200), (220), (311) and (222) planes of fcc Au (JCPDS 04-0784), respectively. The diffraction peaks of Fe_3_O_4_ became weak in Au/Fe_3_O_4_, which may be due to the heavy atom effect of Au coating on the Fe_3_O_4_ supports^20^,^21^. For PdAu/Fe_3_O_4_ nanocomposite, besides the characteristic peaks of Fe_3_O_4_, the (111) and (200) peaks of PdAu (2*θ* = 38.24° and 44.48°) locate between those of monometallic fcc Pd and Au (Fig. 2b), again indicating the formation of bimetallic alloy^22^,^23^.

**Figure 3:**
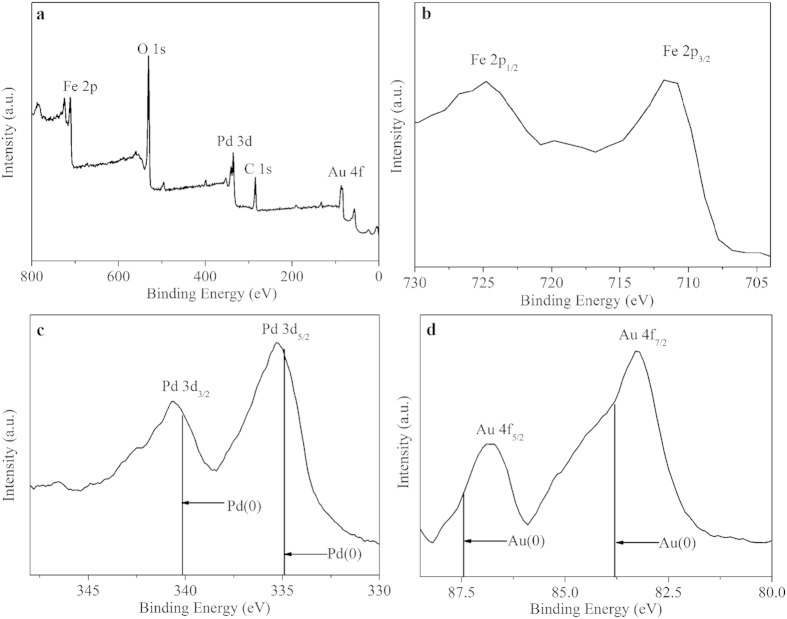
XPS spectra of PdAu/Fe_3_O_4_. (**a**) survey scan, (**b**) Fe 2p, (**c**) Pd 3d and (**d**) Au 4f.

X-ray photoelectron spectroscopy (XPS) was used to characterize the electronic properties and chemical state information of PdAu/Fe_3_O_4_ nanocomposites. Figure 3a reveals the presence of not only Pd and Au, but also Fe, O and C elements from Fe_3_O_4_ and residual cellular and organic components. The binding energies of Fe 2p_3/2_ and Fe 2p_1/2_ are 711.5 eV and 724.5 eV, respectively, which correspond well with those of bulk Fe_3_O_4_ (Fig. 3b). The Pd 3d and Au 4f spectra show that the binding energies of both Pd 3d (3d_5/2_ = 335.3 eV; 3d_3/2_ = 340.8 eV) and Au 4f (4f_7/2_ = 83.5 eV; 4f_5/2_ = 87.1 eV) slightly deviate from the standard values of bulk Pd(0) (3d_5/2_ = 334.9 eV; 3d_3/2_ = 340.2 eV) and bulk Au(0) (4f_7/2_ = 83.8 eV; 4f_5/2_ = 87.5 eV) (Fig. 3c,d). The decrease in Au binding energy and the increase in Pd binding energy for the PdAu/Fe_3_O_4_ nanocomposites suggest the perturbed electronic interaction between Pd and Au atomic orbit and electron transfer from Pd to Au metal during alloy formation^24^. The depletion in electrons could make Pd easier to interact with catalytic reactants.

**Figure 4:**
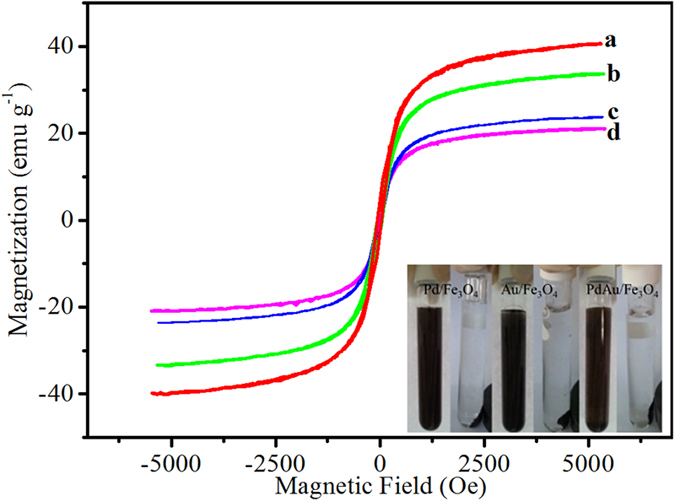
Magnetic properties. Magnetic hysteresis loops of (**a**) Fe_3_O_4_, (**b**) Pd/Fe_3_O_4_, (**c**) Au/Fe_3_O_4_ and (**d**) PdAu/Fe_3_O_4_. The insert pattern showed the magnetic separation of Pd/Fe_3_O_4_, Au/Fe_3_O_4_ and PdAu/Fe_3_O_4_ after catalysis reaction.

The magnetic properties of the obtained nanocomposites were evaluated using vibrating sample magnetometer (VSM) (Fig. 4). The magnetic coercivity or remanence values of biogenic nanocomposites are nearly zero, indicating their superparamagnetic behaviour. The saturation magnetization of biogenic Fe_3_O_4_ (44.34 emu g^−1^) decreased with the addition of non-magnetic noble metal components. However, even the lowest saturation magnetization, which was detected with PdAu/Fe_3_O_4_ (23.63 emu g^−1^) was sufficient to provide an easy and effective separation of the nanocomposite from aqueous solution (Fig. 4 inset).

### Involvement of bound organic components in the formation of Fe_3_O_4_ nanorod and PdAu alloy

**Figure 5:**
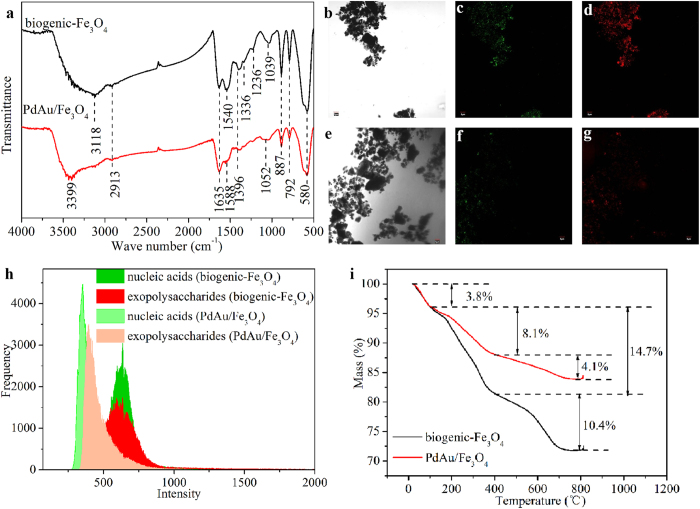
Characterization of the organic components on the surface of different biogenic nanomaterials. (**a**) FTIR spectra. (**b**–**g**) CLSM images. (**b**,**e**) bright-field microscopy, (**c**,**f**) green fluorescence (SYTO9) representing nucleic acids, and (**d**,**g**) orange fluorescence (lectin PHA-L conjugates) representing exopolysaccharides on (**b**–**d**) biogenic Fe_3_O_4_ and (**e**–**g**) PdAu/Fe_3_O_4_. (**h**) fluorescence intensity curves corresponding to CLSM images. (i) TAG analyses of Fe_3_O_4_ and PdAu/Fe_3_O_4_.

The absorption bands of Fourier transform infrared spectroscopy (FTIR) at 887 cm^−1^, 792 cm^−1^ and 580 cm^−1^ were related to the Fe-O bending vibration (Fig. 5a). The absorption peaks at 2913 cm^−1^, 1540–1588 cm^−1^, 1396 cm^−1^, 1236–1336 cm^−1^, and 1039–1052 cm^−1^ were ascribed to fatty acids, amide II, carboxylic groups, amide III and carbohydrates, respectively. Moreover, the absorption peaks at 3118 cm^−1^ and 3399 cm^−1^ correspond to the hydroxyl group and the band at 1635 cm^−1^ was assigned to the bending vibration of water. The intensity of most bands corresponding to organic functional groups weakened or even disappeared after the formation of PdAu/Fe_3_O_4_, implying that some organic components may be consumed during the formation of PdAu alloy.

Confocal fluorescence microscopy (CLSM) analyses were also applied to characterize the organic components on magnetite surfaces. Dark spots of the Fe_3_O_4_ and PdAu/Fe_3_O_4_ were observed with bright-field microscopy (Fig. 5b,e). The intense green fluorescence that was observed after staining with SYTO9 indicates the presence of nucleic acid on the surface of mineral aggregates (Fig. 5c). The results of PHA-L staining show that the biogenic Fe_3_O_4_ nanoparticles are associated with or surrounded by a significant amount of exopolysaccharides (Fig. 5d). Much less intensive fluorescence was observed with PdAu/Fe_3_O_4_ nanoparticles stained with SYTO9 and PHA-L (Fig. 5f,g), which further suggests the consumption of these organic components during the formation of PdAu alloy nanoparticles.

Three significant weight loss steps can be observed in the thermogravimetric (TGA) analysis of biogenic Fe_3_O_4_ and PdAu/Fe_3_O_4_ (Fig. 5i). The first at temperatures lower than 100 °C is due to the dehydration of samples. Around 14.7% and 8.1% weight losses were detected for biogenic Fe_3_O_4_ and PdAu/Fe_3_O_4_ during the second step from 100 to 400 °C, which may be due to the thermal decomposition of adsorbed organic substances. And around 10.4% and 4.1% weight losses were found for the two samples during the third step from 400 to 800 °C, which could be ascribed to the further decomposition of organic components included in the samples.

After mixing the biogenic Fe_3_O_4_ nanoparticles with Pd and Au precursor salts, time-course TEM images of the mixture were recorded. As shown in Supplementary Fig. S6, nanorod structure with length of 36–60 nm and width of 4–8 nm appeared at 10 h and grew as time went on. After 24 h, the length and width of the nanorod increased to 40–75 nm and 8–10 nm, respectively. Finally, the nanorod was 200–300 nm in length and 8–18 nm in width in 48 h (see Supplementary Fig. S3). HRTEM analysis indicates an angle of 35° between (511) and the cross section of nanorod (see Supplementary Fig. S5), which suggests that the nanorod grows along the [220] direction. When alkaline-washed Fe_3_O_4_, which lost most of its organic functional groups (as confirmed by FTIR analysis in Supplementary Fig. S7), was mixed with Pd and Au precursor salt solutions, only nanoparticles with an average diameter of 9.9 ± 2.2 nm and no rod-like structure were observed in the resultant products (see Supplementary Fig. S8). Moreover, EDX analysis of the resultant nanocomposite detected no Pd signal (see Supplementary Fig. S8). And no Pd or PdAu peak but only Au peaks were observed in XRD data (see Supplementary Fig. S8). These results indicate that the organic components are vital for the appearance and growth of Fe_3_O_4_ nanorod and formation of PdAu alloy on Fe_3_O_4_ supports.

### Catalytic reduction of nitroaromatics

Time-dependent UV-vis absorption spectra were monitored throughout the 4-nitrophenol (4-NP) reduction process in the absence or presence of different nanocomposites (see Supplementary Fig. S9). Although NaBH_4_ is a strong reductant, very little decrease of the absorbance at 400 nm (which was assigned to 4-nitrophenolate anions) was observed in 16 min without addition of catalyst. Limited decrease of absorbance at 400 nm was observed in reaction system added with Au/Fe_3_O_4_ nanocomposite. When Pd/Fe_3_O_4_, PdAu/Fe_3_O_4_ or the physical mixture of Pd/Fe_3_O_4_ and Au/Fe_3_O_4_ (Pd/Fe_3_O_4_ + Au/Fe_3_O_4_, with equal elemental Pd and Au masses as in PdAu/Fe_3_O_4_) were added into the reaction system, along with the decrease of the absorbance at 400 nm, a new absorption peak at 300 nm corresponding to 4-aminophenol (4-AP) appeared and increased gradually. Moreover, the appearance of two isosbestic points that locate at 280 nm and 314 nm suggest that only one product is formed during the reaction. About 94.0 ± 1.0% reduction of 4-NP was observed with system provided with PdAu/Fe_3_O_4_ in 8 min, whereas reduction efficiencies of 24.5 ± 2.6%, 76.4 ± 1.6% and 76.1 ± 0.4% were obtained in systems added with Au/Fe_3_O_4_, Pd/Fe_3_O_4_ and Pd/Fe_3_O_4_ + Au/Fe_3_O_4_, respectively.

**Figure 6:**
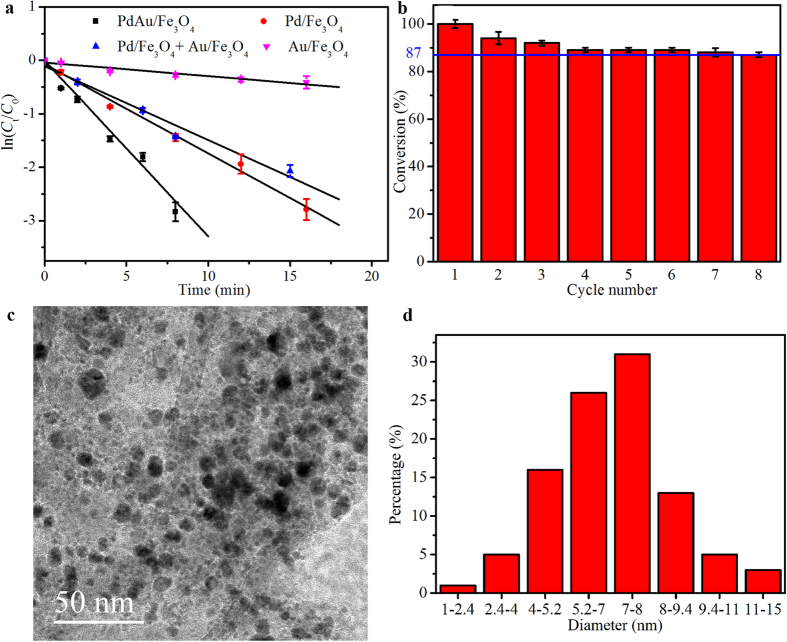
Catalytic performances. (**a**) Plots of ln(*C*_t_/*C*_0_) versus time for the reduction of 4-NP by NaBH_4_ in the presence of Pd/Fe_3_O_4_, Au/Fe_3_O_4_, Pd/Fe_3_O_4_ + Au/Fe_3_O_4_ or PdAu/Fe_3_O_4_. (**b**) The reusability of PdAu/Fe_3_O_4_ as catalyst for reduction of 4-NP by NaBH_4_. (**c**,**d**) TEM image and size distribution of PdAu/Fe_3_O_4_ after reusing for eight runs. Error bars represented standard deviation (n = 3). Significant differences based on the one-way ANOVA (*p* < 0.05).

Since NaBH_4_ was present in great excess in the reduction system, the reaction rate was almost independent of its concentration. Thus the reaction kinetics can be evaluated by a pseudo-first-order process with respect to the concentration of 4-NP. Typical plots of ln(*C*_t_/*C*_0_) against the reaction time (t) for different catalysts were shown in Fig. 6a, where *C*_t_ and *C*_0_ are the 4-NP concentrations at time t and 0, respectively. The values of apparent kinetic rate constant *k*_*app*_ that estimated from linear regression of experimental data for Au/Fe_3_O_4_, Pd/Fe_3_O_4_ and Pd/Fe_3_O_4_ + Au/Fe_3_O_4_ are 0.0255 ± 0.0033 min^−1^, 0.1671 ± 0.0075 min^−1^ and 0.1382 ± 0.0133 min^−1^, respectively. In comparison, the *k*_*app*_ value for PdAu/Fe_3_O_4_ was calculated to be 0.3282 ± 0.0229 min^−1^, which is almost 2-, 2- and 13-fold larger than those of Pd/Fe_3_O_4_, Pd/Fe_3_O_4_ + Au/Fe_3_O_4_ and Au/Fe_3_O_4_, respectively. Activity parameter *k*_Pd_ = *k*_*app*_/*M*_Pd_, where the apparent rate constant *k*_*app*_ is divided by the concentration of Pd (mg l^−1^), was used for a quantitative evaluation and comparison of the catalytic activity of Pd-containing nanocatalysts. As shown in Table 1, the catalytic activity of the biogenic PdAu/Fe_3_O_4_ nanocomposite is comparable to or even better than those of some previously reported counterparts synthesized by chemical methods.

Recycling is important for noble metal-based catalysts in practice, therefore the reusability of PdAu/Fe_3_O_4_ was investigated. As shown in Fig. 6b, the magnetic alloy nanoparticles can be readily recovered and reused for at least eight successive cycles with conversion efficiencies higher than 87%. The *k*_*app*_ values of PdAu/Fe_3_O_4_ gradually decreased with the increase of cycle numbers (see Supplementary Table S1), which may be due to the accumulation and inhibition effects of reaction products. However, after eight rounds of recycling usage, the *k*_*app*_ value of PdAu/Fe_3_O_4_ (0.1937 ± 0.0111 min^−1^) is still higher than those of Au/Fe_3_O_4_ (0.0255 ± 0.0033 min^−1^) and Pd/Fe_3_O_4_ (0.1671 ± 0.0075 min^−1^) used for the first run. PdAu/Fe_3_O_4_ nanoparticles and nanorods can still be seen in TEM image after eight runs of catalysis (Fig. 6c). A little decease of their sizes (an average diameter of 6.7 ± 2.3 nm for nanoparticles, 80–180 nm in length and 8–16 nm in width for nanorods) may indicate the slight loss of nanocatalysts during repeated catalysis.

Besides 4-NP, seven other nitroarenes including nitrobenzene, 2-nitrotoluene, 3-nitrotolune, 4-nitrotoluene, 2-nitrophenol, 3-nitrophenol and 4-nitrochlorobenzene were also used as substrates to test and compare the catalytic activities of Pd/Fe_3_O_4_, Au/Fe_3_O_4_ and PdAu/Fe_3_O_4_. All the nitroaromatic compounds investigated could be reduced to different extents by NaBH_4_ in the presence of a small amount of Pd/Fe_3_O_4_, Au/Fe_3_O_4_ and PdAu/Fe_3_O_4_ (see Supplementary Table S2). For nitrotoluenes, complete reduction was achieved in 47 min in systems added with PdAu/Fe_3_O_4_, whereas more than 71.7 ± 2.3% and less than 14.1 ± 2.5% reduction were achieved in the presence of Pd/Fe_3_O_4_ and Au/Fe_3_O_4_, respectively. It took systems added with PdAu/Fe_3_O_4_ 180 min to reach 77.9 ± 2.3% to 99.7 ± 1.1% reduction of nitrophenols. Higher reduction extent was observed with *m*-nitrophenol over *o*- and *p*-nitrophenol in the presence of Pd/Fe_3_O_4_, whereas almost no difference was observed in reduction efficiencies of different nitrophenols when Au/Fe_3_O_4_ was used. Among the three nanocomposites tested, PdAu/Fe_3_O_4_ generally demonstrates the highest *k*_*app*_ values for nitroaromatic substrates studied (see Supplementary Fig. S10 and Table S3).

## Discussion

Microbe plays a key role in biotransformation and geochemical cycling of redox-active elements in natural environment and can be harnessed for applications in bioremediation and biotechnology. Biopreparation of nanomaterials has attracted a lot of attention during past years due to its environment-friendliness and cost-effectiveness^5^,^6^. *Shewanella* strains can effectively generate, adsorb to and utilize naturally occurring and anthropogenic nanosized Fe oxides, noble metals, metalloids and TiO_2_, and carbon nanotube and graphene etc^7^,^8^,^9^,^25^,^26^,^27^,^28^. Therefore, applying *Shewanella* to synthesize magnetically recyclable noble metal nanocatalysts under ambient conditions deserves investigation.

Monometallic Pd or Au and bimetallic PdAu alloy on magnetite supports were synthesized through sequential incubation of MR-1 cells with akaganeite and Pd/Au salts as precursors. Characterization results of HRTEM, elemental mapping, XRD and XPS confirmed the successful preparation of Pd/Fe_3_O_4_, Au/Fe_3_O_4_, and PdAu/Fe_3_O_4_ nanocomposites. Although there have been several reports on the synthesis and application of biogenic noble metal nanoparticles, the reclamation and repeated use of such materials remain unsolved^11^,^12^. Coker *et al*. reported that *Geobacter sulfurreducens* can reduce Fe(III)-oxyhydroxide to magnetite with the help of anthraquinone-2,6-disulfonate (AQDS). Then the biomagnetite was functionalized with palladium nanoparticles to catalyze Heck reaction^17^. Our study here avoids the use of AQDS, which is a pollutant itself when released into environment and can increase the production cost. Moreover, for the first time, PdAu alloy is biologically produced and immobilized on magnetite supports, which further demonstrates the great capacity of microbial cells for nanomatieral synthesis.

**Figure 7:**
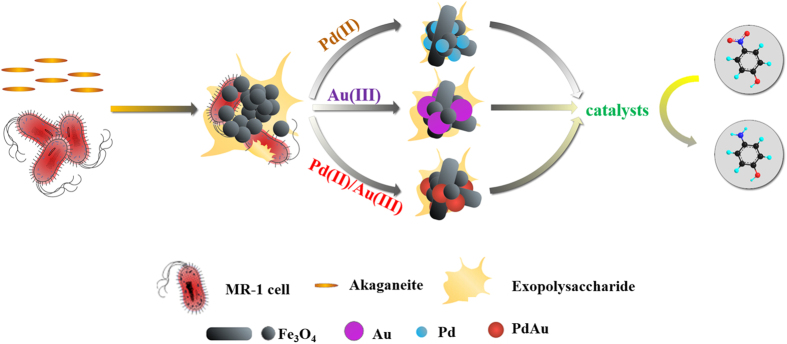
Scheme for synthesis and application of Pd/Fe_3_O_4_, Au/Fe_3_O_4_ and PdAu/Fe_3_O_4_ nanoparticles. The biogenic Fe_3_O_4_ nanoparticles were firstly produced by *S. oneidensis* MR-1 from akaganeite, and then served as support for the further synthesis of Pd, Au and PdAu nanoparticles from respective precursor salts. Microbially originated organic substances like surface-bound cellular components and exopolysaccharides not only function as shape-directing agent to convert some Fe_3_O_4_ nanoparticles to nanorods, but also participate in the formation of PdAu alloy nanoparticles on magnetite. The catalytic capabilities of the resultant nanocomposites were tested with the reduction of 4-NP by NaBH_4_.

It has been reported that microbial extracellular polymeric substance could function as nucleation core or template for the formation of various metal(loid) nanomaterials^29^,^30^. Moreover, it was suggested that the c-type cytochromes contained in extracellular polymeric substance of *S. oneidensis* might be involved in electron transfer and serve as extracellular sites for reducing U(VI) to UO_2_ nanoparticles^31^,^32^. On the surface of biogenic Fe_3_O_4_ nanoparticles, FTIR, CLSM and TGA analyses identified the presence of various organic substances, which may originate from residual/lysed cells and cell-excreted extracellular polymeric substance. Compared to that of biogenic Fe_3_O_4_ nanoparticles, disappearance of most FTIR absorption bands, less intensive fluorescence and lower weight loss were detected with PdAu/Fe_3_O_4_ nanocomposite, indicating the consumption of extracellular polymeric substance during precipitation of PdAu alloy nanoparticles on magnetite surface (Fig. 7). Therefore, the formation of PdAu/Fe_3_O_4_ nanocomposite may require the presence of surface-bound organic components.

The presence of these microbially originated organic substances on Fe_3_O_4_ surface avoids the need of precoating Fe_3_O_4_ nanoparticles with organic ligand or silica shell, which are generally required for the following adsorption and immobilization of noble metal on the magnetic support^33^,^34^. Moreover, the reduction potential of Au(III) (*E*^0^_AuCl4_^−^_/Au_ = +1.00 V *vs*. SHE) is higher than that of Pd(II) (*E*^0^_PdCl4_^2−^_/Pd_ = +0.6 V *vs*. SHE)^35^. Therefore, Au(III) is preferentially reduced by microbial components and extracellular polymeric substance (*E*^*0*^ = −0.32 to −0.1 V *vs*. NHE)^36^ over Pd(II) when both of them are simultaneously exposed to biogenic Fe_3_O_4_ nanoparticles, resulting in higher content of Au than Pd in PdAu/Fe_3_O_4_. Surprisingly, Au nanoparticles were still formed on alkaline-washed Fe_3_O_4_ without the help of organic components. Previous studies have suggested that magnetite can reduce Hg(II), Np(V), U(VI) and Se(IV) to their lower-valence or even elementary states^37^,^38^,^39^,^40^. Thus magnetite may be responsible for the reduction of Au(III) to Au(0) in this situation. However, the organic components are needed for the formation of Pd or PdAu nanoparticles on biogenic Fe_3_O_4_.

In addition, results of control experiments showed that the presence of Fe_3_O_4_-associated organic substances was required for the generation of rod-like magnetite after the addition of noble metal precursor salts. It has been reported that, in the presence of externally added organic substances, Fe_3_O_4_ nanoparticles can self-assemble into nanorods, nanowires or nanosheets without temple through the interplay and balance of dipolar force, electrostatic interaction and van der Waals force^16^,^41^,^42^. The self-assembly of Fe_3_O_4_ nanoparticles into oriented nanosheets was achieved through using a hydrophilic terpolymer as stabilizer under low pH conditions^42^. Jiang *et al*. utilized bio-inspired dopamine to help the growth of Fe_3_O_4_ nanoparticles into nanowires^16^. Organic components from microbial cells may serve as stabilizer and shape-directing agent to facilitate the growth and formation of Fe_3_O_4_ nanorods.

The catalytic capabilities of the biosynthesized nanocomposites were tested with the reduction of 4-NP into 4-AP in the presence of excessive NaBH_4_. The reaction has been widely applied as a benchmark to test the catalytic ability of various nanocatalysts. Differentiated catalytic activities of these biogenic nanocomposites (PdAu/Fe_3_O_4_ > Pd/Fe_3_O_4_ > Au/Fe_3_O_4_) were found during 4-NP reduction. Remarkably, the *k*_Pd_ value of the biogenic PdAu/Fe_3_O_4_ for 4-NP reduction is comparable with or even higher than those of some chemically synthesized Pd-based catalysts. Pd is the main component responsible for the catalytic activity of the biogenic nanocomposites. The introduction of Au and formation of PdAu alloy significantly improve the catalytic activity of nanocomposites. However, simply physical mixing of Pd/Fe_3_O_4_ and Au/Fe_3_O_4_ did not result in enhanced catalytic activity when compared with Pd/Fe_3_O_4_. The improved catalytic activity of alloyed PdAu nanoparticles compared to that of monometallic Pd nanoparticles has been attributed to geometric and electronic effects after the introduction of Au, which can cause a contraction of the lattice and withdraw electron density from Pd (as also suggested by the XPS data)^12^,^43^.

The same order of catalytic activity, i.e. PdAu/Fe_3_O_4_ > Pd/Fe_3_O_4_ > Au/Fe_3_O_4_, was observed in the reduction of some other nitroaromatic substrates. The reduction efficiencies of nitrophenols are generally lower than those of nitrobenzene and nitrotoluene compounds. Although both methyl and hydroxyl are electron donating groups, the higher electron-donating property of hydroxyl group leads to less positively charged nitrogen, the attachment of which to the negatively charged hydrogen from the Pd metal-hydrogen structure is hindered. The position of substitute groups also impacts the reduction activity of nitroaromatic compounds. For all the three kinds of nanocatalysts, the reduction activities of hydroxyl- and methyl-substituted nitrobenzenes (i.e. nitrophenols and nitrotoluenes) generally follow a descending order of *meta*-substituted > *ortho*-substituted > *para*-substituted, which can be explained by conjugation and inductive effects. For both nitrophenols and nitrotoluenes, the stability of the nitro group was increased by the delocalization of the negative charge throughout the benzene ring into it. On the other hand, the inductive effects of *ortho*- and *meta*-substituted groups could destabilize the substituted nitrocompounds. The inductive effect of *ortho*-substituted group is less effective due to its steric hindrance. And *meta*-substituted group has only inductive effect but no conjugate effect. Thus the *meta*-substituted nitrocompound is the least stable among the three isomers^44^.

In summary, we have demonstrated a facile and efficient route for synthesizing Pd, Au and PdAu alloy on biogenic Fe_3_O_4_ nanoparticles/nanorods by *S. oneidensis* MR-1. Microbial extracellular polymeric substances binding on the surfaces of biogenic Fe_3_O_4_ participate in the appearance of rod-like Fe_3_O_4_ and formation of PdAu/Fe_3_O_4_ nanocomposite. Excellent catalytic activities towards the reduction of different nitroaromatic compounds were observed with the prepared nanocomposites, among which PdAu/Fe_3_O_4_ demonstrated the highest catalytic activity and satisfying stability. The present findings may open up a new and environmentally benign avenue in the development of magnetic noble metal nanocomposites.

## Methods

### Strain and culture conditions

*S. oneidensis* MR-1 was routinely cultured in Luria-Bertani broth medium aerobically overnight at 30 °C under shaking conditions (150 rpm). Then the cell culture was harvested by centrifugation (11000 *g*, 5 min) and washed three times with piperazine-N,N′-bis(2-ethanesulfonic acid) (PIPES) buffer (20 mM, pH 7.0).

### Synthesis of Fe_3_O_4_ nanoparticles

Akaganeite precursor was synthesized according to a previously described method^18^. Briefly, 10 M NaOH was slowly added into 0.4 M FeCl_3_·6H_2_O solution under stirring conditions until the pH reached 7.0. The suspension was allowed to ripen for 6–8 h, washed thrice with Milli-Q water (18.2 MΩ·cm) and then resuspended in N_2_-flushed Milli-Q water followed by anaerobic capping.

The washed cells were resuspended in anaerobic PIPES buffer to a final concentration of 1.39 g l^−1^. Akaganeite (40 mM) and lactate (10 mM) were added as electron acceptor and donor, respectively. The bio-reduction system was anaerobically incubated in the dark at 30 °C for microbial synthesis of magnetite nanoparticles, the appearance of which can be detected by permanent magnet.

### Synthesis of Pd/Fe_3_O_4_, Au/Fe_3_O_4_ and PdAu/Fe_3_O_4_ nanocomposites

After 48 h incubation, the biosynthesized Fe_3_O_4_ nanoparticles were harvested, washed three times with degassed Milli-Q water and separated from the supernatant using external magnet.

The biogenic Fe_3_O_4_ nanoparticles were then resuspended in degassed Milli-Q water in serum bottles to reach a final concentration of 0.8 mM. To synthesize Pd/Fe_3_O_4_ or Au/Fe_3_O_4_ nanocomposites, Na_2_PdCl_4_ or HAuCl_4_ was added from degassed stock solutions to the serum bottles to reach a final concentration of 1 mM. Both of the two precursor salts were added simultaneously into the serum bottles (each at a final concentration of 1 mM) to synthesize PdAu/Fe_3_O_4_ nanocomposite. Lactate (10 mM) was supplemented as electron donor. The serum bottles were anaerobically incubated in the dark at 30 °C for 48 h. Then the resultant nanoparticles were collected by external magnet and washed three times with degassed Milli-Q water. Finally, the harvested nanomaterials were resuspended in degassed Milli-Q water before further characterization and activity test.

To study the effects of Fe_3_O_4_-associated organic substances on the appearance and growth of Fe_3_O_4_ nanorods and formation of PdAu alloy, the harvested biogenic Fe_3_O_4_ nanoparticles were treated with 0.5 M NaOH at 30 °C for 24 h under shaking conditions (150 rpm) to remove the absorbed organic substances, and then washed with degassed Milli-Q water through centrifugation for several times until the pH of the supernatant reached neutral.

### Characterization

Pd(II), Au(III) and Fe(III) concentrations were measured with a Perkin-Elmer 200-DV inductively coupled plasma optical emission spectrometer. TEM and EDX analysis were performed on Tecnai G2 Spirit TEM operating at 120 kV. HRTEM images and elemental mapping were obtained using a NOVA nanosem 450 HRTEM at 300 kV. XRD was measured with a D/max-2400 diffractometer using CuK radiation (λ = 0.1541 nm). Electronic binding energies were measured by a Thermo Scientific K-Alpha XPS. FTIR spectra were taken in KBr pressed pellets with an EQUINOX55 FTIR. The magnetization curves of nanocomposite samples were measured with a JDM-13 VSM. TGA was carried out on a TGA-DTA6300 instrument at a heating rate of 10 °C min^−1^ up to a final temperature of 800 °C in a nitrogen flow (20 ml min^−1^).

CLSM observation was performed by using FLUOVIEW FV1000MPE microscope equipped with an Ar-ion laser (488 nm) and a HeNe-laser (543 nm). Samples were stained in the dark for 10 min with 383 μg ml^−1^ SYTO9, a dye that stains Gram-negative bacteria nucleic acids (green fluorescence), and 50 μg ml^−1^ lectin PHA-L conjugates for exopolysaccharide (orange fluorescence).

### Catalytic reduction of nitroaromatic compounds

In a typical experiment, aqueous 4-NP solution (5 ml, 200 mg l^−1^) and freshly prepared NaBH_4_ solution (5 ml, 1.6 g l^−1^) were mixed in a glass vial. Immediately after the addition of PdAu/Fe_3_O_4_ suspension (1.13 μg ml^−1^ Pd in the reaction system) under shaking conditions (150 rpm), the 4-NP reduction reaction was monitored using UV-vis spectroscopy in a scanning range of 200–600 nm. The catalytic activities of Pd/Fe_3_O_4_ and Au/Fe_3_O_4_ nanocomposite were also tested following similar procedures at the same concentrations of Pd or Au (1.13 μg ml^−1^ in the reaction system), respectively. Moreover, the catalytic activity of Pd/Fe_3_O_4_ + Au/Fe_3_O_4_ mixture (with the same final masses of elemental Pd and Au referred to those of PdAu/Fe_3_O_4_) for the reduction of 4-NP was also measured.

In the recycle test of the catalytic activity of PdAu/Fe_3_O_4_, after the solution became colorless, which indicated the accomplishment of the reaction, another 50 μl mixture of 4-NP (20 g l^−1^) and 8 mg NaBH_4_ were directly added into the reaction mixture for the next run. This step was repeated for seven rounds to study the stability of the catalysts.

The reduction of other nitroaromatics including nitrobenzene, 2-nitrotoluene, 3-nitrotolune, 4-nitrotoluene, 2-nitrophenol, 3-nitrophenol and 4-nitrochlorobenzene was also studied with Pd/Fe_3_O_4_, Au/Fe_3_O_4_ and PdAu/Fe_3_O_4_. Each kind of nanocomposite was added into mixture of 5 ml aqueous solutions of different nitroaromatic compounds (200 mg l^−1^) and 5 ml freshly prepared NaBH_4_ solution (1.6 g l^−1^) in a glass vial (0.0644 μg Pd or Au ml^−1^ in the reaction system). The reduction process was monitored at intervals by high performance liquid chromatography with a UV detector and C18 column (Hypersil ODS-2, 5 mm, 4.6*250 mm).

### Statistical analysis

All experiments were performed at least three times and the data were shown as mean ± standard deviation. The normality of the nanoparticle size distribution was determined by the Kolmogrov-Smirnov test. Differences in catalytic reduction of nitroaromatic compounds by Pd/Fe_3_O_4_, Au/Fe_3_O_4_ or PdAu/Fe_3_O_4_ were compared by a one-way analysis of variance (ANOVA) and *p*-value of <0.05 was considered significant. The data were analyzed using SPPS 19.0.

## Additional Information

**How to cite this article**: Tuo, Y. *et al*. Microbial synthesis of Pd/Fe3O4, Au/Fe3O4 and PdAu/Fe3O4 nanocomposites for catalytic reduction of nitroaromatic compounds. *Sci. Rep*. **5**, 13515; doi: 10.1038/srep13515 (2015).

## Supplementary Material

Supplementary Information

## Figures and Tables

**Table 1:** Catalytic rate constants of different nanocatalysts for 4-NP reduction.

Catalyst	Pd concentration (M_Pd_, mg l^−1^)	*k*_*app*_ (min^−1^)	*k*_Pd_ (l min^−1^ mg^−1^)	Reference
PdAu/Fe_3_O_4_	1.13	0.3282	0.2904	This work
Pd/Fe_3_O_4_	1.13	0.1671	0.1479	This work
Au-Pd carbon spheres	22.67	0.8760	0.0386	45
Pd@Au core-shell nanotetrapods	1.05	0.1390	0.1324	46
Pd/Ag dendrites	13.52	2.3460	0.1734	47
Ni@Pd core–shell nanoparticles	13.16	1.2240	0.0930	48
Fe_3_O_4_@C/Pd	40.00	0.1950	0.0049	49
Pd/Fe_3_O_4_/polypyrrole	0.94	0.1220	0.1298	50
