# Supplementary material for: Microbial synthesis of Pd/Fe3O4, Au/Fe3O4 and PdAu/Fe3O4 nanocomposites for catalytic reduction of nitroaromatic compounds
Source: Sci Rep. 2015 Aug 27;5:13515. doi: 10.1038/srep13515 (PMC4550933; doi:10.1038/srep13515)
Supplement: Supplementary Information [file srep13515-s1.pdf]

Supplementary Information

Microbial synthesis of Pd/Fe_3O_4, Au/Fe_3O_4 and PdAu/Fe_3O_4 nanocomposites for catalytic reduction of nitroaromatic compounds

Ya Tuo[1], Guangfei Liu[1, *], Bin Dong[1], Jiti Zhou[1], Aijie Wang[2, *], Jing Wang[1], Ruofei Jin[1], Hong Lv[1], Zeou Dou[1], Wenyu Huang[1]

[1] Key Laboratory of Industrial Ecology and Environmental Engineering, Ministry of Education, School of Environmental Science and Technology, Dalian University of Technology, Dalian, 116024, China

[2] State Key Laboratory of Urban Water Resource and Environment, Harbin Institute of Technology, Harbin, 150090, China

*corresponding authors:

Guangfei Liu, guangfeiliu@dlut.edu.cn

Aijie Wang, waj0578@hit.edu.cn

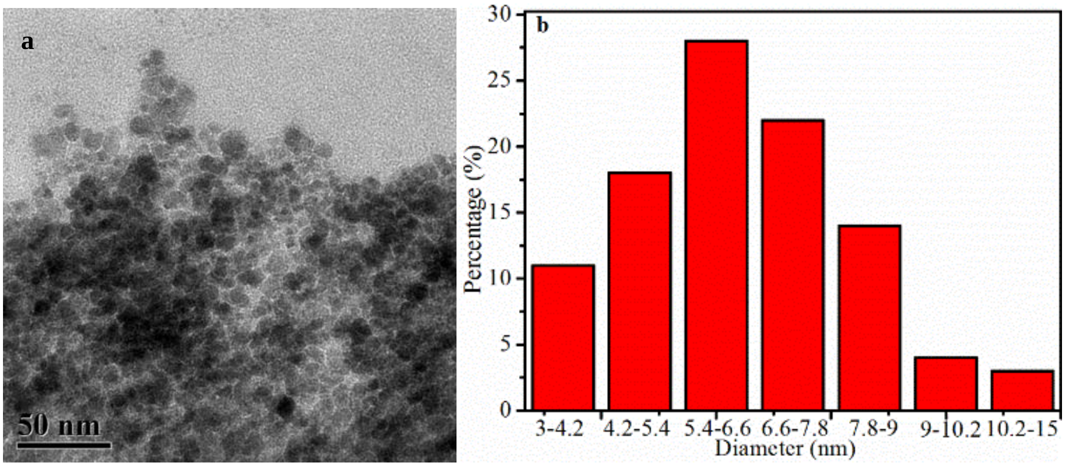

Fig. S1 Morphology and size distribution of biogenic Fe_3O_4 nanoparticles. (a) TEM image and (b) size distribution. Test of normality of nanoparticles size was determined by the Kolmogorov-Smirnov test.

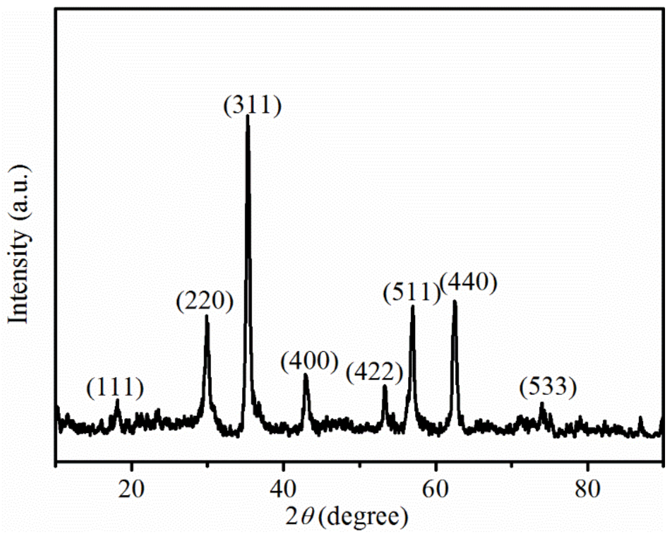

Fig. S2 XRD analysis of biogenic Fe_3O_4 nanoparticles. The Fe_3O_4 nanoparticles were collected by external magnet, washed three times with the degassed Milli-Q water and then dried under vacuum at 60 ℃ for XRD analysis.

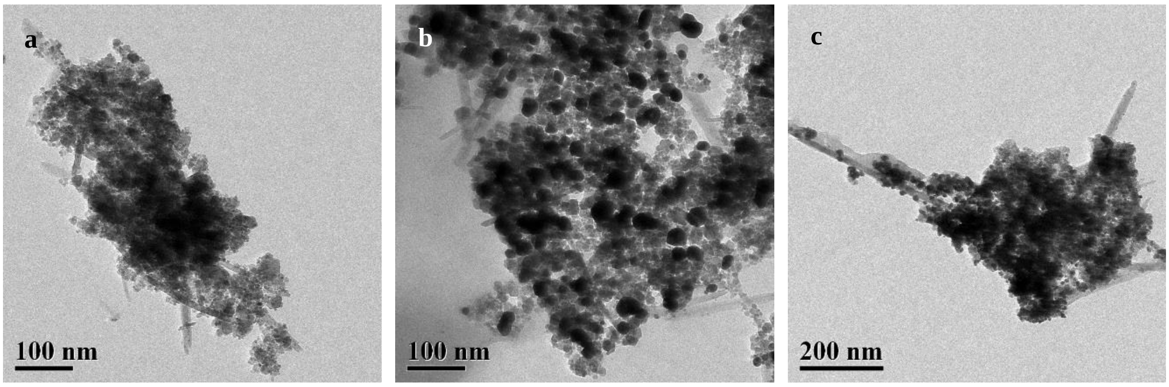

Fig. S3 TEM images of different nanocomposites after 48 h incubation of biogenic Fe_3O_4 with noble metal precursor salt solutions. (a) Pd/Fe_3O_4, (b) Au/Fe_3O_4 and (c) PdAu/Fe_3O_4

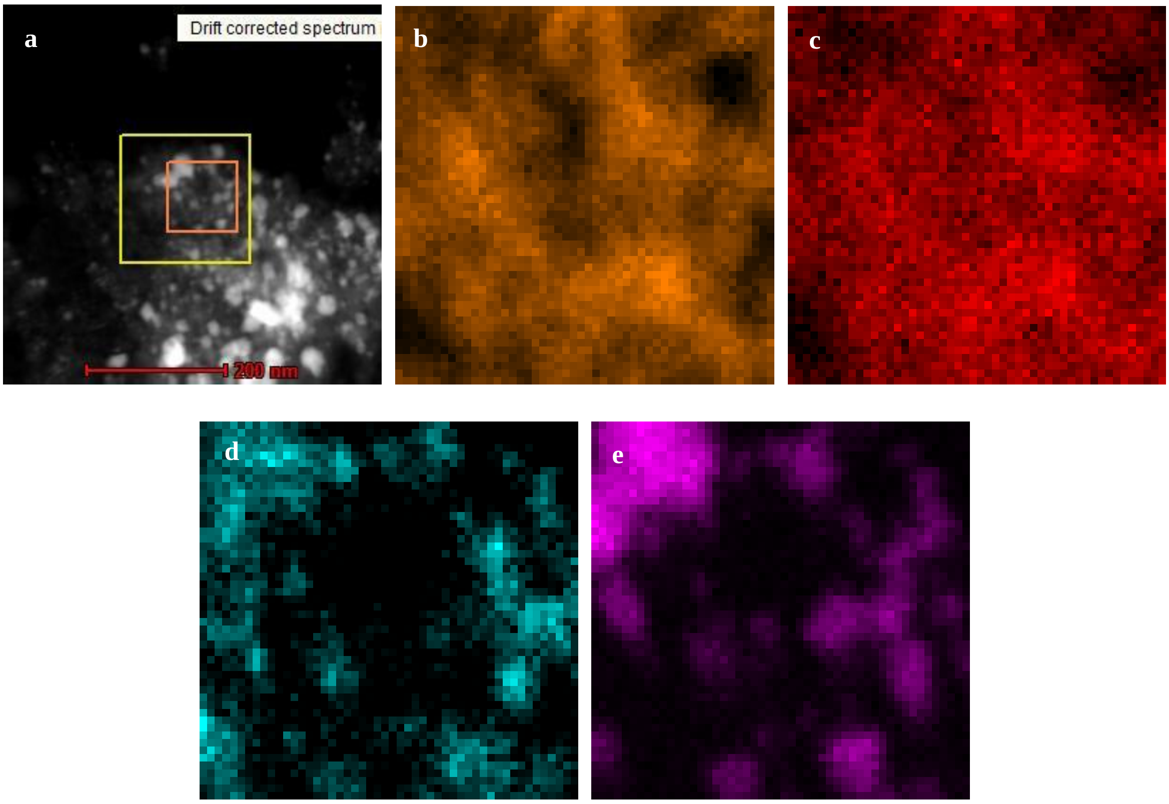

Fig. S4 EDX mapping analysis of PdAu/Fe_3O_4. (a) Dark-field TEM image, (b) Fe, (c) O, (d) Pd and (e) Au.

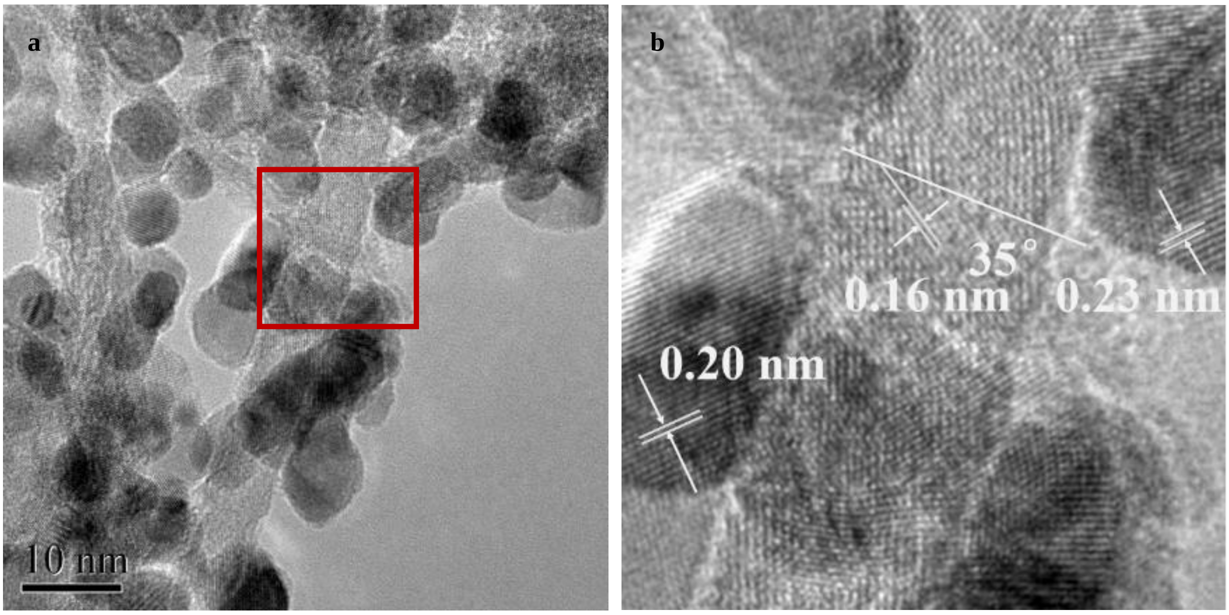

Fig. S5 Characterization of PdAu/Fe_3O_4. (a) HRTEM image and (b) the related magnification section.

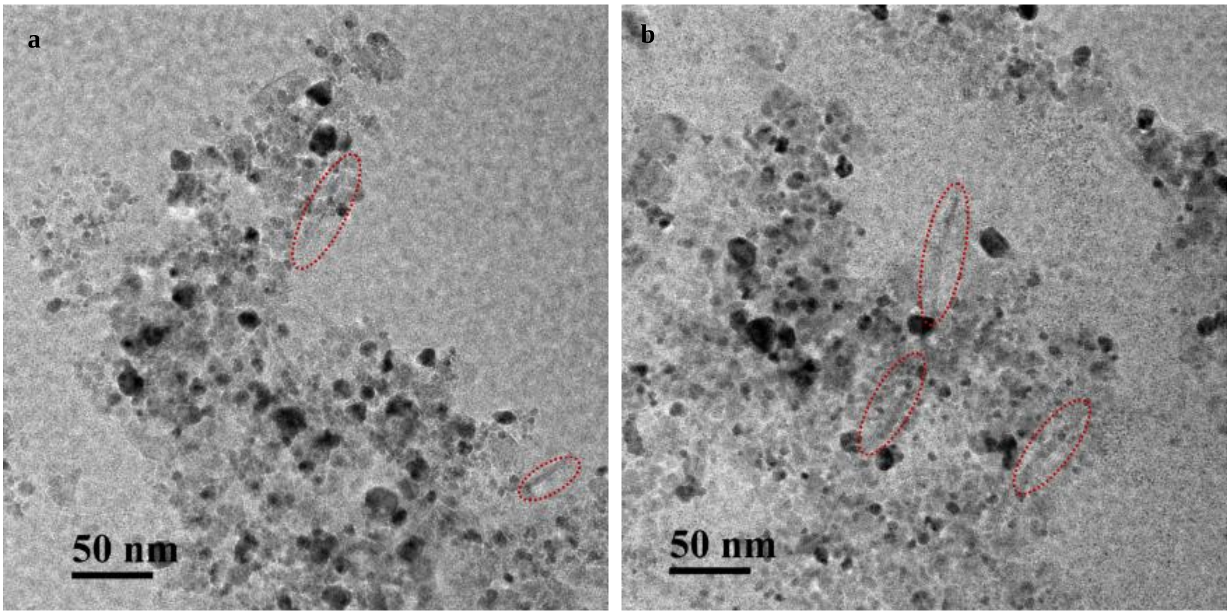

Fig. S6 Time-course TEM images of PdAu/Fe_3O_4. (a) 10 h and (b) 24 h.

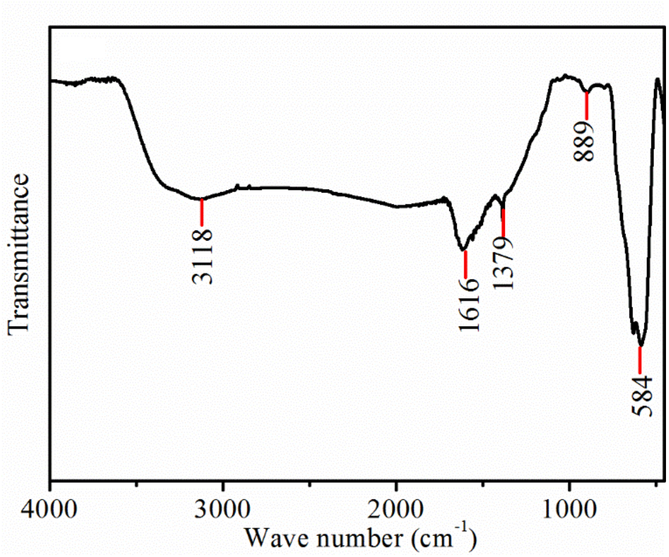

Fig. S7 FTIR analysis of the alkaline-washed Fe_3O_4. The biogenic Fe_3O_4 nanoparticles were treated with NaOH to remove the absorbed organic substances, and then washed for several times until the pH of the supernatant reached neutral.

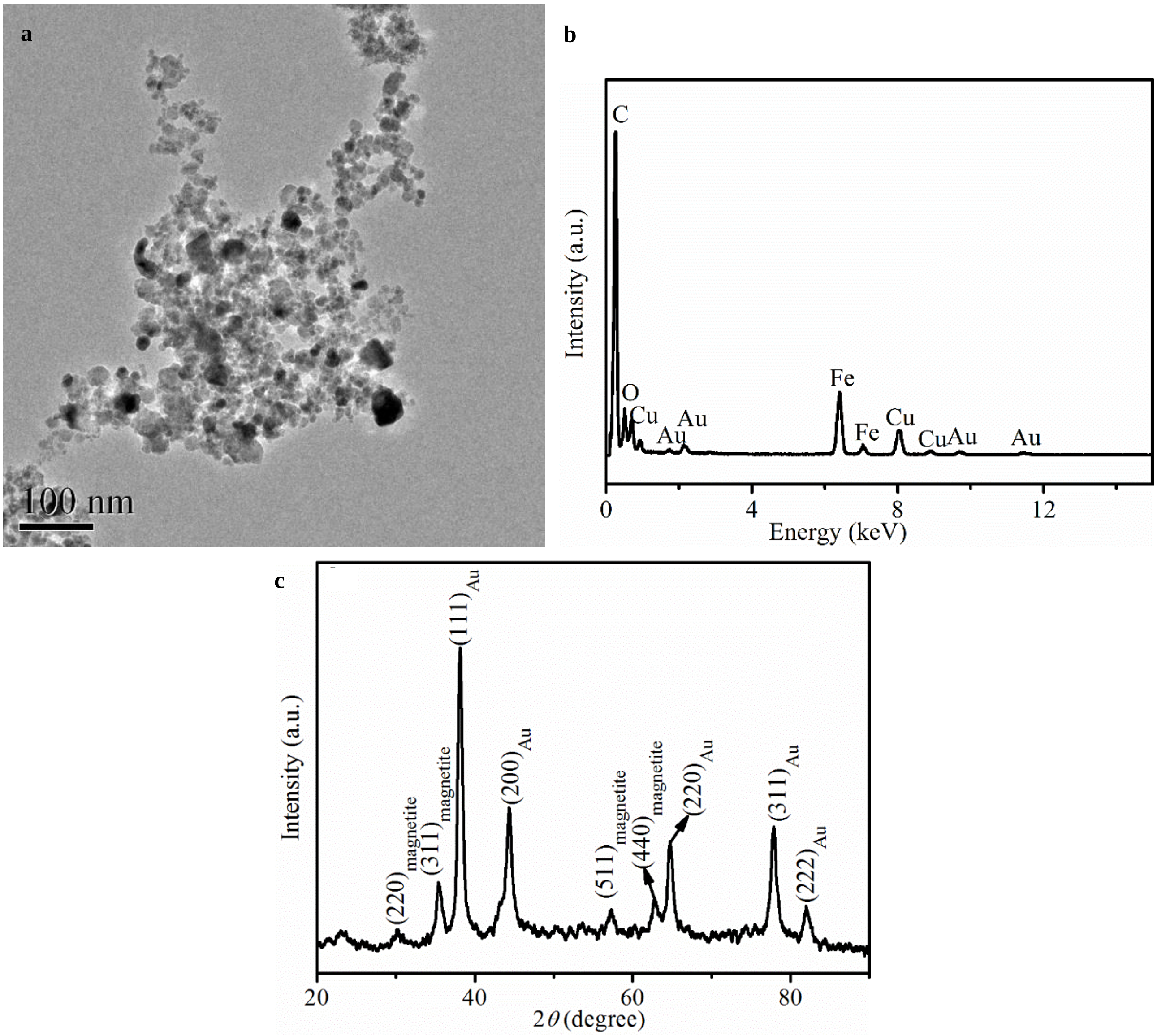

Fig. S8 Characterization of the nanomaterials synthesized on alkaline-washed Fe_3O_4 after mixing with Pd and Au precursor salt solutions. (a) TEM image, (b) EDX and (c) XRD pattern.

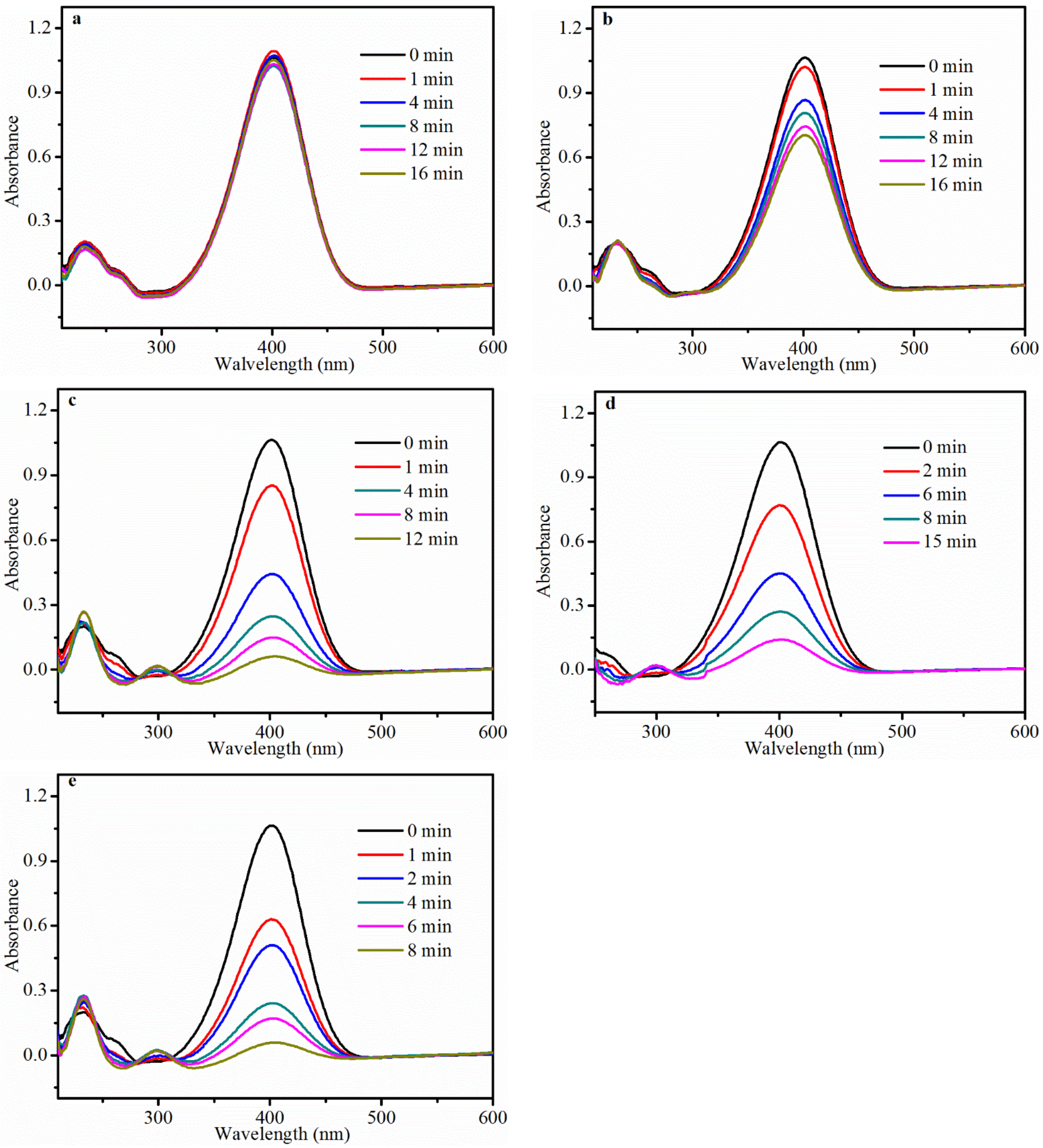

Fig. S9 Catalytic performances. Time-dependent UV–vis spectra of 4-NP reduction by $NaBH_4$ in the presence of (a) no catalyst, (b) Au/Fe_3O_4, (c) Pd/Fe_3O_4, (d) Pd/Fe_3O_4+Au/Fe_3O_4 and (e) $PdAu/Fe_3O_4$.

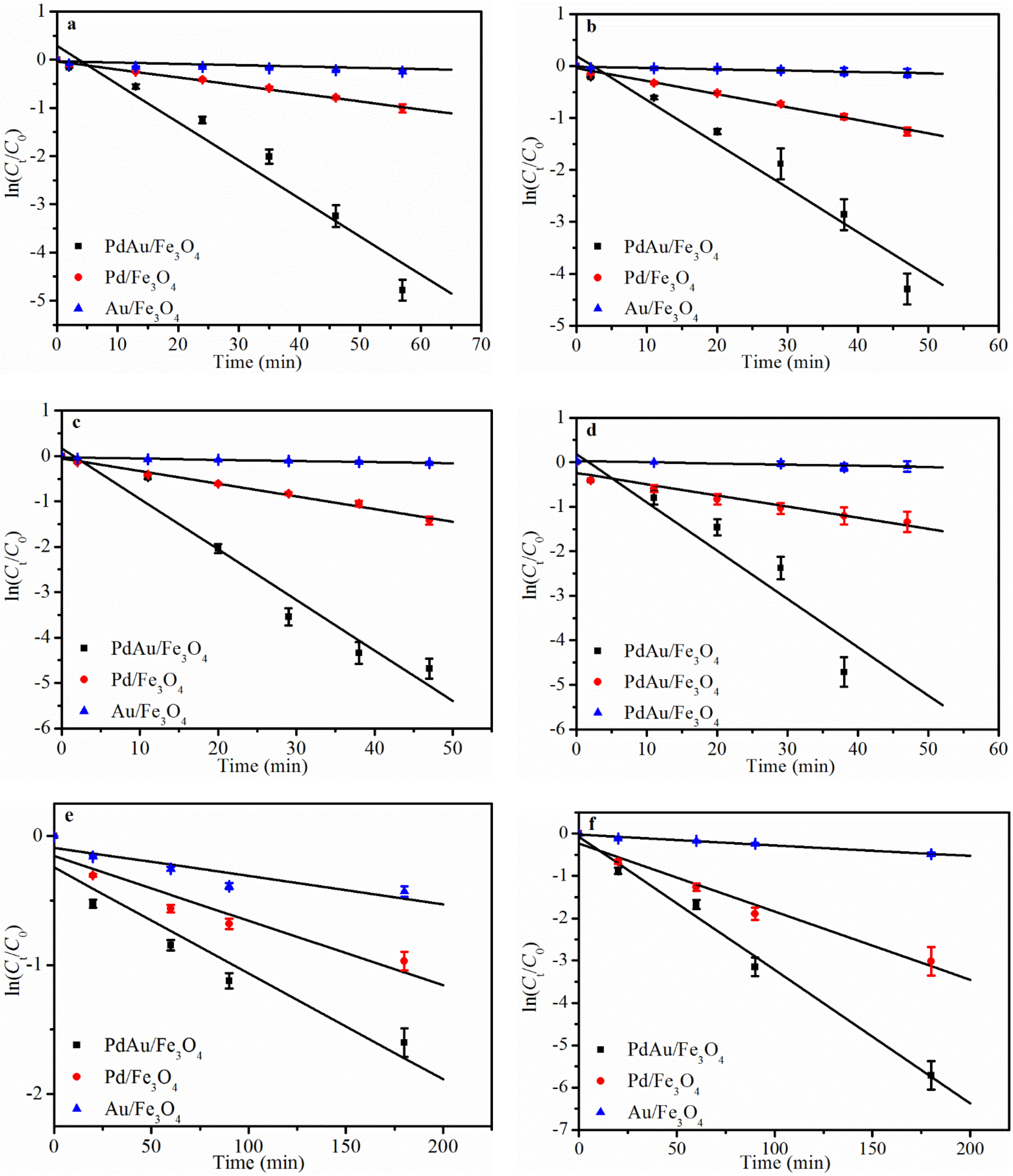
a
ln(C_t/C_0)
Time (min)
PdAu/Fe3O4
Pd/Fe3O4
Au/Fe3O4
b
ln(C_t/C_0)
Time (min)
PdAu/Fe3O4
Pd/Fe3O4
Au/Fe3O4
c
ln(C_t/C_0)
Time (min)
PdAu/Fe3O4
Pd/Fe3O4
Au/Fe3O4
d
ln(C_t/C_0)
Time (min)
PdAu/Fe3O4
PdAu/Fe3O4
PdAu/Fe3O4
e
ln(C_t/C_0)
Time (min)
PdAu/Fe3O4
Pd/Fe3O4
Au/Fe3O4
f
ln(C_t/C_0)
Time (min)
PdAu/Fe3O4
Pd/Fe3O4
Au/Fe3O4

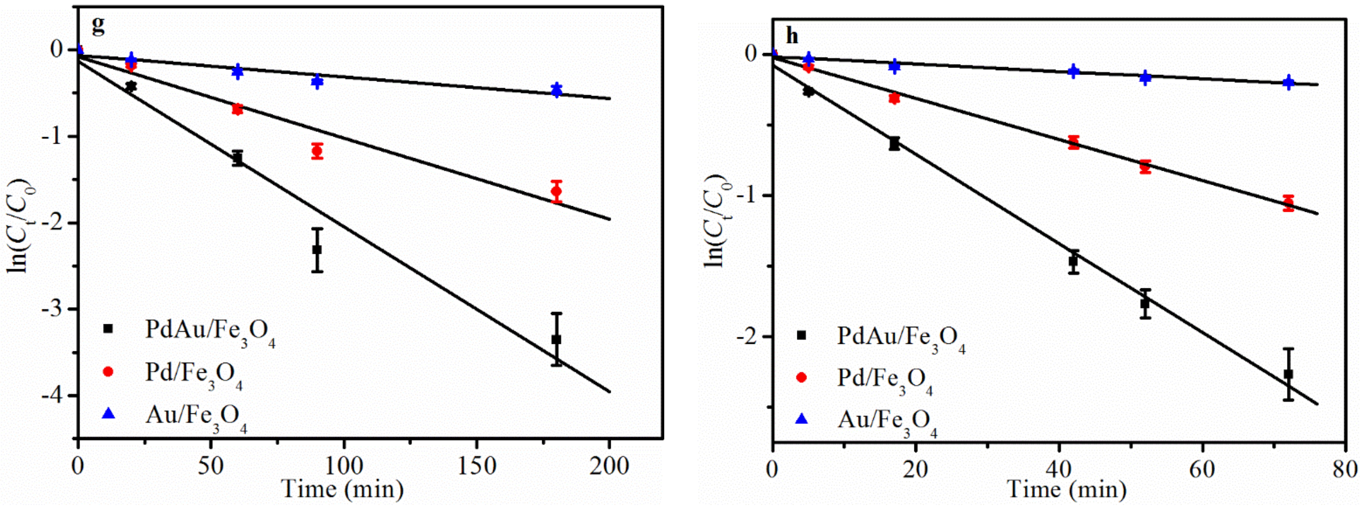

Fig. S10 Kinetics analyses. Plots of ln(C_t/C_0) versus time for the reduction of (a) nitrobenzene, (b) 4-nitrotoluene, (c) 3-nitrotolune, (d) 2-nitrotolune, (e) 4-nitrophenol, (f) 3-nitrophenol, (g) 2-nitrophenol and (h) 4-nitrochlorobenzene. Error bars represent standard deviation (n=3). Significant differences based on the one-way ANOVA (p<0.05).

Table S1 Apparent kinetic constant (k_{app}) values of PdAu/Fe_3O_4 in recycling runs of 4-NP reduction. The k_{app} values represent the mean ± deviation (n=3). Significant differences based on the one-way ANOVA (p<0.05)

Recycling run	1	2	3	4	5	6	7	8
k_{app} (min^{-1})	0.3282 ± 0.0229	0.2852 ± 0.0151	0.2418 ± 0.0186	0.2323 ± 0.0053	0.2292 ± 0.0038	0.2239 ± 0.0089	0.1976 ± 0.0076	0.1937 ± 0.0111

Table S2 Comparison of reductive conversion efficiency of different nitroaromatics when using Pd/Fe_3O_4, Au/Fe_3O_4 and $PdAu/Fe_3O_4$ as catalysts [%conversion attained during reaction time (min)]. Conversions represent the mean ± deviation (n=3). Significant differences based on the one-way ANOVA ($p<0.05$)

Entry	Starting nitroaromatics	Product	Pd/Fe_3O_4		Au/Fe_3O_4		$PdAu/Fe_3O_4$	
			Time (min)	conversion %	Time (min)	conversion %	Time (min)	conversion %
1	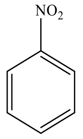	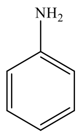	57	63.6 ±2.9	57	22.1 ±2.6	57	99.2 ±0.6
2	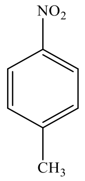	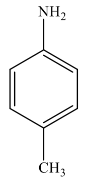	47	71.7 ±2.3	47	12.6 ±1.9	47	98.6 ±0.5
3	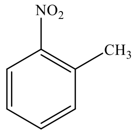	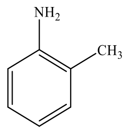	47	73.9 ±2.9	47	12.4 ±2.2	47	100 ±0.1
4	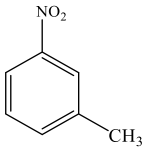	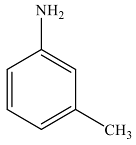	47	75.8 ±2.3	47	14.1 ±2.5	47	99.1 ±0.2
5	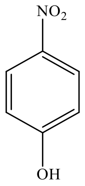	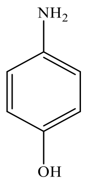	180	62.1 ±2.5	180	35.0 ±2.5	180	79.9 ±2.3
6	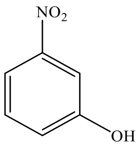	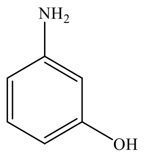	180	95.1 ±1.5	180	38.2 ±2.3	180	99.7 ±1.1
7	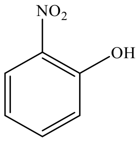	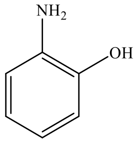	180	80.6 ±2.3	180	37.2 ±2.6	180	96.5 ±1.1
8	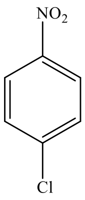	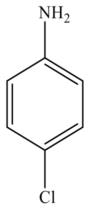	72	65.2 ±1.7	72	18.1 ±1.1	72	89.6 ±1.8

Table S3 The k_{app} values of reducing different nitroaromatics in the presence of Pd/Fe_3O_4, Au/Fe_3O_4 and $PdAu/Fe_3O_4$. The k_{app} values represent the mean ± deviation (n=3). Significant differences based on the one-way ANOVA (p<0.05)

Substrate	k_{app} (min^{-1})		
	$PdAu/Fe_3O_4$	Pd/Fe_3O_4	Au/Fe_3O_4
nitrobenzene	0.0790 ± 0.0078	0.0166 ± 0.0007	0.0034 ± 0.0007
4-nitrotoluene	0.0848 ± 0.0076	0.0251 ± 0.0010	0.0025 ± 0.0002
3-nitrotoluene	0.1113 ± 0.0093	0.0279 ± 0.0012	0.0026 ± 0.0004
2-nitrotoluene	0.1086 ± 0.0178	0.0254 ± 0.0032	0.0030 ± 0.0005
4-nitrophenol	0.0082 ± 0.0014	0.0050 ± 0.0009	0.0022 ± 0.0006
3-nitrophenol	0.0315 ± 0.0017	0.0162 ± 0.0015	0.0025 ± 0.0002
2-nitrophenol	0.0191 ± 0.0022	0.0094 ± 0.0013	0.0025 ± 0.0004
4-nitrochlorobenzene	0.0315 ± 0.0011	0.0145 ± 0.0004	0.0026 ± 0.0002
